# End‐stage renal disease in a child with focal segmental glomerulosclerosis associated with a homozygous *NUP93* variant

**DOI:** 10.1002/ccr3.5111

**Published:** 2021-11-16

**Authors:** Ratna Acharya, Kiran Upadhyay

**Affiliations:** ^1^ Division of General Pediatrics Department of Pediatrics University of Florida Gainesville Florida USA; ^2^ Division of Pediatric Nephrology Department of Pediatrics University of Florida Gainesville Florida USA

**Keywords:** child, focal segmental glomerulosclerosis, nephrotic syndrome, *NUP93*

## Abstract

This report highlights that the genetic causes of FSGS, including *NUP93* gene variant, such as the one described in this report, progress to end‐stage renal disease rapidly and that the risk of recurrence post‐renal transplantation is less likely.

## Graphical abstract

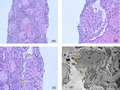

## 1 INTRODUCTION

Several podocyte gene variants are associated with steroid‐resistant nephrotic syndrome. We describe an 8‐year‐old girl who presented with nephrotic range proteinuria and was found to be homozygous in the nucleoporin (*NUP93*) gene for a novel sequence variant. This is the first case report description of such novel variant.

The monogenic causes of nephrotic syndrome (NS) are a rapidly evolving field with the discovery of new podocyte genes. These podocytopathies account for about one‐quarter of pediatric steroid‐resistant nephrotic syndrome (SRNS) cases.^1^ Also, unlike immune‐mediated NS, most patients with inherited podocytopathies are more likely to have rapid progression to end‐stage renal disease (ESRD).^2^ The gene affected and the type of variant strongly determine the age at first presentation and the rate of disease progression.^2^ Hence, a genetic diagnosis is helpful not only for better understanding of the disease but also to assess therapeutic success of immunosuppressive agents, and to predict the risk of recurrence after transplantation.^3^

Nucleoporins (NUP) are nuclear envelope proteins that form the nuclear pore complex (NPC) and are located in the inner (*NUP93* and *NUP205*) and outer (*NUP85*, *NUP107*, *NUP133*, and *NUP160*) ring of the NPC. NUPs play roles in the nucleocytoplasmic transport, SMAD signaling, chromatin organization, regulation of gene expression, and DNA repair.^4^ Recently, a few cases of SRNS due to *NUP93* variants have been reported.^5^, ^6^ Here, we report a homozygous *NUP93* novel sequence variant in an Indian child with focal segmental glomerulosclerosis (FSGS) leading to rapidly progressive ESRD. This variant is predicted to result in the amino acid substitution p. Ile714Thr and has not been reported in literature or public databases.

## 2 CASE PRESENTATION

A previously healthy eight‐year‐old girl who was born in India and migrated to the United States at four years of age presented with hypertensive urgency and nephrotic range proteinuria (urine protein ≥ 300 mg/dl and spot urine protein to creatinine ratio of 14 mg/mg). She was asymptomatic besides headaches. Urine output was normal, and there was no history of facial puffiness or swelling of feet or abdomen. There was no history of usage of non‐steroidal anti‐inflammatory drugs and other nephrotoxic agents, dehydration, and recent infections including urinary tract infections. There were no other known significant past medical problems. She was born full term with no perinatal complications. Family history was significant for consanguinity. On examination, the child was at 75th percentile for height and 55th percentile for weight. Besides strabismus, there was no dysmorphism, periorbital puffiness, ascites, or pedal edema. Gross physical examination of all other organ systems was normal. Pertinent laboratory test showed serum albumin 3.1 gm/dl, blood urea nitrogen 14 mg/dl, and serum creatinine 1.6 mg/dl. Serum total cholesterol was mildly elevated (200 mg/dl) with normal HDL and LDL cholesterol, and normal triglycerides. Blood counts were normal. There was no microscopic hematuria. Serum complements were normal. Antinuclear and anti–double‐stranded DNA antibodies were negative. Hepatitis panel, human immunodeficiency virus, and tuberculin test were all negative. Non‐contrast computed tomography scan of the head was normal. Renal bladder sonogram showed bilateral echogenic kidneys with right kidney of 8.2 cm (28th percentile for age, 0.58 standard deviation below the mean) and left kidney of 8.7 cm in length (53rd percentile for age, 0.09 standard deviation above the mean) without hydronephrosis. Nasopharyngeal swab for respiratory viruses including SARS‐CoV‐2 was negative. Echocardiogram showed evidence of mild left ventricular hypertrophy but no other abnormalities.

**FIGURE 1:**
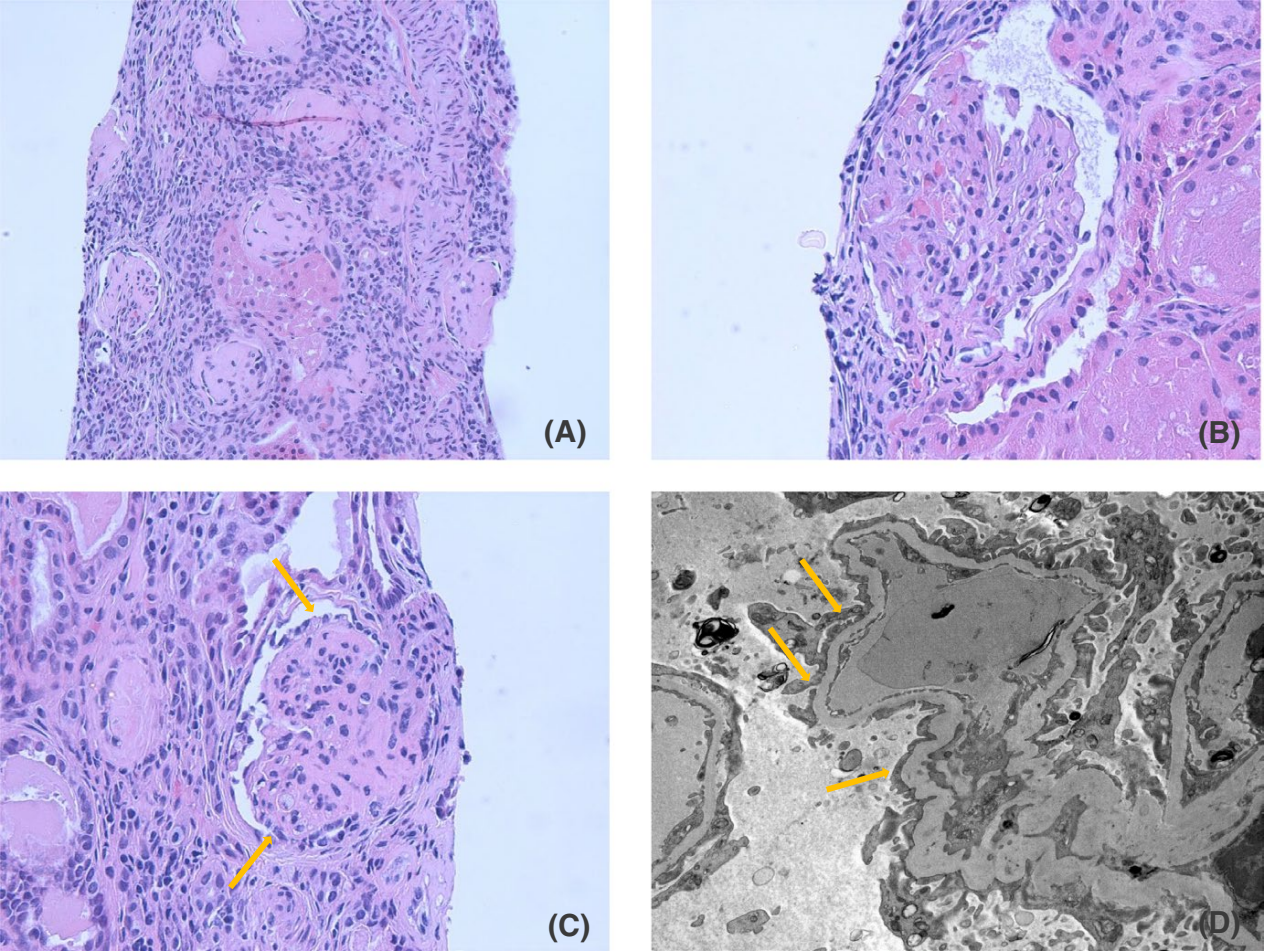
Renal biopsy findings: (A) A majority of glomeruli are globally sclerotic by light microscopy (H&E stain 10× 10). (B) One of remaining viable glomerulus shows normal in mesangial cellularity and matrix (H&E stain 40× 10). (C) Another glomerulus has sclerosis in more than half of glomerulus capillary tuft, associated with podocyte hyperplasia (H&E stain 40× 10). (D) Podocyte foot processes effacement detected by Electron microscopy (Direct Mag: 8,000)

Serum creatinine over next few days increased to 1.8 mg/dl (Schwartz estimated glomerular filtration rate 32 ml/1.73 m^2^/min). Serum intact parathyroid hormone was 217 pg/ml. and there was mild iron deficiency. Nephrotic range proteinuria persisted but the spot urine protein to creatinine ratio decreased to values ranging from 5 to 7 mg/mg after addition of lisinopril. To determine the acute versus chronic nature of her kidney disease and for diagnostic evaluation of the nephrotic range proteinuria and elevated serum creatinine, a percutaneous renal biopsy was performed which showed 18 out of 20 globally sclerotic glomeruli. One glomerulus showed segmental sclerosis without collapse in majority of glomerular capillary tuft, associated with podocyte hyperplasia and foam cells. There was severe interstitial fibrosis and tubular atrophy (IF/TA). Electron microscopy showed partial foot process effacement without immune deposits (Figure 1A–D). A diagnosis of advanced FSGS, not otherwise specified with diffuse global glomerulosclerosis and severe IF/TA was made. Based upon these findings, although FSGS was the likely cause of nephrotic range proteinuria, given the chronicity observed in the biopsy, it was difficult to establish a direct cause and effect relationship between FSGS and these findings.

Due to the elevated serum creatinine and features of advanced chronic kidney disease in the biopsy, it was likely that the child will require dialysis and/or kidney transplantation in the future. Hence, a genetic testing was performed to determine the genetic risk of recurrence post‐transplantation, and to assess whether immunosuppressive therapy is warranted to manage the nephrotic proteinuria. A combination of next‐generation sequencing (NGS) and sanger sequencing was done to cover the full coding regions of the 72 genes known to cause NS/FSGS along with ~10 bases of non‐coding DNA flanking each exon (Prevention Genetics, Marshfield, WI, USA). The test showed the patient to be heterozygous in the *ACTN4* (NM_004924.5), *INF2* (NM_022489.3), and *KANK1* (NM_015158.3) genes for three novel sequence variants of uncertain significance (c.158G>T, c.3632G>T, and c.2884G>A, respectively). None of these variants are listed in the ClinVar. She was also found to be homozygous in the *NUP93* (NM_014669.4) gene for a novel sequence variant c.2141T>C, which is predicted to result in substitution, transition, missense point mutation leading to replacement of protein isoleucine by protein threonine (p. Ile714Thr) (Table 1). This variant is also not listed in the ClinVar. There were no copy number variants detected within the genomic regions of this test with a sensitivity approaching 100%. Although ~10 bases of non‐coding DNA flanking each exon were analyzed by this test, the deep introns were not analyzed. No further in vitro functional studies were performed; and hence, the exact functional implications of these variants were unknown. However, to determine the pathogenicity of these variants, in silico tests were performed using SIFT (Sorting Intolerant From Tolerant), FATHMM (Functional Analysis Through Hidden Markov Models), PolyPhen‐2 (Polymorphism Phenotyping V‐2), and MutationTaster. The resulted predictions utilizing these *in silico* tools for these four variants were “conflicting” for *ACTN4*, *INF2*, and *NUP93* and “tolerated” for *KANK1* (Table 1). Both parents were also heterozygous carriers of this *NUP93* sequence variant and did not have proteinuria. Similarly, targeted testing of *ACTN4* and *INF2* genes of parents showed that father was heterozygous in the *ACTN4* and *INF2* genes for familial variants of uncertain significance (VUS) designated c.158G>T and c.3632G>T (in silico prediction “conflicting” for both variants), respectively (same as the child). Mother was also heterozygous in the *ACTN4* gene for a familial sequence VUS designated c.158G>T (in silico prediction “conflicting,” same as the child), but did not have the sequence variant *INF2* c.3632G>T (p. Arg1211Leu), as seen in the father and child. There were no other family members with the same condition. Due to inherited gene variants and likelihood of steroid resistance, she was not treated with steroid or immunosuppressive agents. Hypertension was managed with lisinopril, amlodipine, and labetalol with stabilization of blood pressures. She subsequently progressed to ESRD ten months later and was started initially on chronic hemodialysis followed by peritoneal dialysis. She received a deceased donor renal transplant 14 months later with no occurrence of recurrent disease during her most recent follow‐up six months post‐transplant. Her maintenance immunosuppression consisted of tacrolimus, mycophenolate, and prednisone.

**TABLE 1:** Molecular genetics report of Nephrotic syndrome/Focal segmental glomerulosclerosis panel of the patient

Gene, Transcript	Mode of inheritance, Gene OMIM	DNA variations, predicted effects, zygosity	ClinVar ID	Highest allele frequency in a gnomAD population	In Silico Missense Predictions	Interpretation
*ACTN4*, NM_004924.5	AD, 604638	c.158G>T, p. Arg53Leu, Heterozygous	Undocumented	0.0057%, South Asian	Conflicting	UNCERTAIN
*INF2*, NM_022489.3	AD, 610982	c.3632G>T, p. Arg1211Leu, Heterozygous	Undocumented	0.068%, South Asian	Conflicting	UNCERTAIN
*KANK1*, NM_015158.3	AR, 607704	c.2884G>A, p. Ala962Thr, Heterozygous	Undocumented	0.004%, European (Non‐Finnish)	Tolerated	UNCERTAIN
*NUP9*3, NM_014669.4	AR, 614351	c.2141T>C, p. Ile714Thr, Homozygous	Undocumented	Not present	Conflicting	UNCERTAIN

Note

ClinicVar ID: Variant accession (www.ncbi.nlm.nih.gov/clinvar); GnomAD: Allele frequency registered in a large population database (gnomad.broadinstitute.org); value listed is the highest allele frequency reported within one of seven population categories recognized in gnomAD V.2.0. Missense Predictions: Summarized output (Damaging, Conflicting, or Tolerated) via Polyphen‐2, SIFT, MutationTaster, and FATHMM.

Abbreviations: AD, autosomal dominant; AR, autosomal recessive.

## 3 DISCUSSION

Steroid‐resistant nephrotic syndrome is the second most common cause of ESRD in children and young adults.^1^ About 15%–20% of all idiopathic pediatric NS are steroid resistant with prognosis varying from permanent remission to progression to ESRD.^1^ SRNS could be either immune‐mediated or secondary to podocyte gene variants. More than 50 monogenic causes of SRNS have been described so far. Sadowski et al.^7^ showed that monogenic podocytopathies were found in about one‐third of pediatric SRNS. In this study, a single‐gene variant was found in 29.5% of children and young adults with the highest detection in those with SRNS onset in the first three months of life (69.4%) and least if the onset was between 13 and 18 years of age (10.8%).^7^ A causative variant was found in 49.5% of consanguineous families but in only 25% of non‐consanguineous families. Nucleoporin 93 (*NUP*93) was not tested in this study. Our patient was born to consanguineous parents of Indian origin and presented at eight years of age with nephrotic range proteinuria and was homozygous in the *NUP93* gene for a novel sequence VUS along with heterozygous in the *ACTN4*, *KANK1*, and *INF2* genes for novel sequence VUS. Hence, this report adds a novel *NUP93* variant to the genetic spectrum in Indian children with SRNS.

Variants in nucleoporin *NUP93* were recently described as one of the causes of SRNS which can cause rapidly progressive ESRD.^5^, ^6^, ^7^ Braun et al. studied 7 individuals with SRNS (Serbian, German, and Turkish descent) due to recessive *NUP93* variants but none of these individuals had the variant described in this report.^5^ FSGS was the predominant renal histology and one had diffuse mesangial sclerosis. Hematuria was present in some patients; one had Marcus‐Gunn syndrome and rest had no extra‐renal manifestations. One patient responded partially to steroid, and two responded partially to cyclosporine. The age of onset was 1–6 years of age and ESRD occurred at 1–11 years. *NUP93* variant was shown to disrupt NPC assembly and to prevent *NUP93* interaction with the signaling protein SMAD4.^5^ Bezdíčka et al.^6^ reported a 5.4% incidence of *NUP93* variant in children with SRNS from Czech Republic and Slovakia. Seeman et al.^8^ reported recurrent FSGS post‐renal transplant in a 7‐year‐old child with *NUP93* variant. This is in contrast with the common observation of less likelihood of disease recurrence post‐transplant in vast majority of patients with SRNS due to inherited gene mutations.^1^ Sandokji et al. reported a 5‐year‐old non‐consanguineous female of African American and Hispanic origin with *NUP93* variant resulting in ESRD and renal transplant with no recurrence.^9^ We identified a homozygous *NUP93* novel sequence variant in a consanguineous Indian girl who had a rapidly progressive ESRD in the first decade of life with no disease recurrence in the post‐transplant period.

Variants of the outer ring subunit proteins of the NPC can also cause SRNS.^10^, ^11^, ^12^ Park et al.^10^ described Korean children with SRNS caused by *NUP107* variants; all had an earlier onset of NS and more rapid progression to ESRD compared with variant‐negative patient. None of these patients had recurrence of disease after renal transplantation. Braun et al. identified recessive variants in four genes encoding the components of the outer ring subunits of the NPC among 29 individuals of 13 consanguineous families.^11^ FSGS was the primary renal histology. Some of them had extra‐renal manifestations such as short stature, microcephaly, and skeletal/facial phenotypes. The authors showed that knockout of *NUP85*, *NUP107*, or *NUP133* genes in podocytes activated Cdc42 and increased the formation of filopodia which impaired the actin cytoskeleton. Indeed, the dysregulation of the Cdc42 causes impairment of the actin dynamics which plays an important role in the pathogenesis of monogenic SRNS.^13^

Our patient also was heterozygous in the *KANK1* gene for a novel variant. Variants in *KANK* family genes can lead to podocyte dysfunction and autosomal recessive form of NS.^14^ Other heterozygous VUS that were found in our patient were in the *INF2* and *ACTN4* genes; variants affecting function of these genes are well‐known causes of autosomal dominant (AD) SRNS.^3^ Although variants affecting functions of *ACTN4* and *INF2* are transmitted as AD; and hence, heterozygous variants could be potentially causal, the parents who carried the same heterozygous variants of *ACTN4* and *INF2* were asymptomatic without any proteinuria. Hence, given that the child was homozygous for the *NUP93* variant for which parents were heterozygous, the possibility of this *NUP93* variant leading to the nephrotic proteinuria was more likely. However, all of these variants in these genes were reported as VUS; and hence, it is difficult to establish a definite causal relationship between the variant gene and the clinical manifestation. Hence, when such novel variants are reported in a disease context, functional studies, if performed, provide valuable information on how these variants affect the protein function and contribute to the disease phenotype. In our patient, although the functional studies were not performed, in silico analyses were performed in an order to predict the impact of these novel variants in the outcome. However, given the low specificities of these in silico prediction tools, they may not be suitable to predict the pathogenicity of VUS. Hence, it is difficult to derive clinical consequences based solely on in silico predictions. However, one study suggested that these tools could be suitable to predict benignity.^15^ Also, with the advancement of technologies in genetic testing such as NGS, the probability of identification of multiple novel variants in more than one gene is also high. Hence, it is important to interpret the significance of these variants taking into consideration the clinical manifestations, laboratory data, and family history, especially in those with chronic kidney disease of unknown etiology.^16^ Our report also indicates that patients carrying variants in multiple SRNS genes may have more severe disease phenotype, in concordance with prior reported studies.^17^ Lastly, variants in genes not analyzed in this study could be causative of FSGS in this consanguineous girl. Also, since the deep introns were not analyzed, there is a possibility of missing deep intronic variants which have been shown to be associated with FSGS.^18^

## 4 CONCLUSIONS

The discovery of novel genes and their sequence variants in NS helps us understand the molecular mechanisms of disease and predict the disease course and severity. Reports of other cases of *NUP93* variants are needed to improve our understanding of the exact mechanism of podocyte injury in these patients.

## CONFLICT OF INTEREST

The authors declare no conflict of interest.

## AUTHOR CONTRIBUTIONS

RA and KU contributed to conception and design of the study, acquisition and analysis of data, literature review, and drafting the whole manuscript.

## ETHICAL APPROVAL

We testify that: This material has not been published in whole or in part elsewhere; The manuscript is not currently being considered for publication in another journal; We have been personally and actively involved in substantive work leading to the writing of this manuscript and will hold ourselves jointly and individually responsible for its content.

## CONSENT

Written informed consent was obtained from the parent to use the data and publish this report in accordance with journal's patient consent policy.

## Data Availability

The data that support the findings of this study are available from the corresponding author upon reasonable request.
